# Hindered dissolution of fibrin formed under mechanical stress

**DOI:** 10.1111/j.1538-7836.2011.04203.x

**Published:** 2011-05

**Authors:** I Varjú, P Sótonyi, R Machovich, L Szabó, K Tenekedjiev, M M C G Silva, C Longstaff, K Kolev

**Affiliations:** * Department of Medical Biochemistry, Semmelweis UniversityBudapest; † Department of Vascular Surgery, Semmelweis UniversityBudapest; ‡ Chemical Research Center, Hungarian Academy of SciencesBudapest, Hungary; § IT Department, N.Y. Vaptsarov Naval AcademyVarna, Bulgaria; ¶ Biotherapeutics, Haemostasis Section, National Institute for Biological Standards and ControlSouth Mimms, UK

**Keywords:** fibrin, plasmin, plasminogen, shear forces, tPA

## Abstract

**Background:**

Recent data indicate that stretching forces cause a dramatic decrease in clot volume accompanied by gross conformational changes of fibrin structure.

**Objective:**

The present study attempts to characterize the lytic susceptibility of fibrin exposed to mechanical stress as a model for fibrin structures observed *in vivo*.

**Methods and results:**

The relevance of stretched fibrin models was substantiated by scanning electron microscopic (SEM) evaluation of human thrombi removed during surgery, where surface fibrin fibers were observed to be oriented in the direction of shear forces, whereas interior fibers formed a random spatial meshwork. These structural variations were modeled *in vitro* with fibrin exposed to adjustable mechanical stress. After two- and three-fold longitudinal stretching (2 × S, 3 × S) the median fiber diameter and pore area in SEM images of fibrin decreased two- to three-fold. Application of tissue plasminogen activator (tPA) to the surface of model clots, which contained plasminogen, resulted in plasmin generation which was measured in the fluid phase. After 30-min activation 12.6 ± 0.46 pmol mm^−2^ plasmin was released from the non-stretched clot (NS), 5.5 ± 1.11 pmol mm^−2^ from 2 × S and 2.3 ± 0.36 pmol mm^−2^ from 3 × S clot and this hampered plasmin generation was accompanied by decreased release of fibrin degradation products from stretched fibrins. Confocal microscopic images showed that a green fluorescent protein-fusion variant of tPA accumulated in the superficial layer of NS, but not in stretched fibrin.

**Conclusion:**

Mechanical stress confers proteolytic resistance to fibrin, which is a result of impaired plasminogen activation coupled to lower plasmin sensitivity of the denser fibrin network.

## Introduction

Pharmacological thrombolysis is based on administration of plasminogen activators, which attack a solid-phase thrombus from the fluid phase [reviewed in 1]. In the course of this therapy a series of biochemical reactions is initiated, which can be simplified to a two-stage process: activation of plasminogen initially present in the thrombus and digestion of fibrin by the generated plasmin. Because plasminogen activators such as tissue-type plasminogen activator (tPA) bind to fibrin as a prerequisite for efficient plasminogen activation, the structure of the fibrin network affects both stages of this process [reviewed in 2]. Accordingly, in the last decade a number of ultrastructural, viscoelasticity and permeability studies have been carried out in an attempt to identify physical properties of the fibrin in natural thrombi that determine its susceptibility to enzymatic lysis [3–5]. Tighter fiber packing, smaller gel pores, higher fibrin stiffness and lower permeability have all been identified as factors conferring resistance of *ex vivo* thrombi and *in vitro* clots to fibrinolysis. An important advance in our understanding of the physical properties of fibrin as a biopolymer was achieved with the recent report on the molecular and sub-molecular changes in fibrin structure induced by mechanical stretching [6]. Mechanical forces acting in one direction on pre-formed fibrin clots result in longitudinal orientation of the fibers, in which the unfolding of separate domains in the extended single fibrin monomers is accompanied by exposure of hydrophobic domains and expulsion of water out of the fibers with consequent reduction of the fibrin volume. In light of these gross alterations in fibrin structure, the present study was undertaken in an attempt to understand the relationship between clot structure and lytic susceptibility of clots exposed to similar mechanical stress. In addition, we raise the question of the potential biological relevance of similarly altered fibrin structures hypothesizing that the shear forces in stenotic vessels [7] could be sufficiently strong to modify mechanically the fibrin architecture.

## Methods

### Patients

Ten patients (4 men and 6 women, mean age: 66 years; range: 49–91) subjected to thrombectomy were enrolled in the study. Eight of them had obliterative thrombosis localized in large arteries (femoral, ileac, popliteal and brachial) based on atherosclerosis (in four cases the thrombus was in a previously implanted graft). One patient had venous thrombosis and the pulmonary embolizing thrombus was removed, one thrombus was from a resected aorta aneurysm. No inherited or acquired thrombophilic state could be diagnosed in the examined group. At the time of thrombectomy all patients received heparin treatment. Written informed consent was obtained from all patients and the study protocol was approved by the institutional and regional ethical board.

### Scanning electron microscope (SEM) imaging of thrombi and fibrin

Immediately (within 5 min) after the surgery or the preparation of fibrin, 5 × 5 × 10 mm pieces of thrombi or fibrin clots of 100-μL volume were placed into 10 mL 100 mmol L^−1^ Na-cacodylate pH 7.2 buffer for 24 h at 4 °C. After repeated washes with the same buffer, samples were fixed in 1% (v/v) glutaraldehyde for 16 h. The fixed samples were dehydrated in a series of ethanol dilutions [20–96% (v/v)], 1:1 mixture of 96% (v/v) ethanol/acetone and pure acetone followed by critical point drying with CO_2_ in E3000 Critical Point Drying Apparatus (Quorum Technologies, Newhaven, UK). The specimens were mounted on adhesive carbon discs, sputter coated with gold in a SC7620 Sputter Coater (Quorum Technologies) and images were taken with scanning electron microscope EVO40 (Carl Zeiss GmbH, Jena, Germany).

### Preparation of fibrin clots exposed to mechanical stress

Elastic silicon rubber tubes (3 mm internal diameter) were soaked in 25% (v/v) Triton X-100 solution for 1 h and thoroughly washed with water. Human fibrinogen (plasminogen-depleted; Calbiochem, LaJolla, CA, USA) at 30 μmol L^−1^ in 10 mmol L^−1^ HEPES-NaOH 150 mmol L^−1^ NaCl pH 7.4 buffer was clotted in these tubes with 30 nmol L^−1^ thrombin (thrombin from Serva Electrophoresis GmbH [Heidelberg, Germany] was further purified by ion-exchange chromatography on sulfopropyl-Sephadex yielding preparation with specific activity of 2100 IU mg^−1^ [8]) at 37 °C for 30 min. Thereafter 1.5 cm long pieces of fibrin were cut and used for SEM imaging or fibrinolytic measurements as non-stretched (NS fibrin with 106 μL volume and 140 mm^2^ surface area). For fibrinolytic experiments with stretched fibrin, 2.25 or 1.5 cm long pieces of the rubber tubes with fibrin inside were stretched to a final length of 4.5 cm and used as 2 × S fibrin (16 μL volume and 94.7 mm^2^ surface area) and 3 × S fibrin (10.6 μL volume and 77.4 mm^2^ surface area), respectively (the stretching manipulation is illustrated in Fig. S1, supporting information available online). The volume and surface area of fibrin were estimated from the initial dimensions of the rubber mould and the data reported in [6] for the volume changes of stretched fibrin. For SEM imaging and confocal microscopy the fibrin clots were removed from the mould, stretched and kept in this state with compression under the clamps of Bürker chambers during glutaraldehyde fixation or under glass coverslips of self-designed confocal microscopic chambers.

### Expression and characteristics of fluorescent chimeric tPA variant (tPA-GFP)

Recombinant human tPA-jelly fish green fluorescent protein (GFP) was constructed and expressed using the Bac-toBac baculovirus expression system as a tPA-C-terminal fusion with Enhanced Green Fluorescent Protein (EGFP) isolated from the pEGFP plasmid (Clonetech, Mountain View, CA, USA), as described previously [9,10].

### Confocal microscopic imaging in the course of fibrinolysis

Fibrin clots were prepared from 30 μmol L^−1^ fibrinogen containing 50 nmol L^−1^ Alexa Fluor® 546-conjugated fibrinogen (Invitrogen Life Technologies, Budapest, Hungary) and 200 nmol L^−1^ plasminogen (isolated from human plasma [11]), clotted with 30 nmol L^−1^ thrombin in 0.5-mm high chambers constructed from glass slides. Thereafter 60 nmol L^−1^ tPA-GFP was added to the edge of the clot and the fluorescence (excitation wavelength 488 nm, emission wavelength 525 nm for tPA-GFP detection and excitation wavelength 543 nm, emission wavelength 575 nm for Alexa^546^-fibrinogen detection) was monitored with Confocal Laser Scanning System LSM510 (Carl Zeiss GmbH) taking sequential images of the fluid-fibrin interface at a distance of approximately 50 μm from the glass surface with identical exposures and laser intensities.

### Plasminogen activation on fibrin surface

Plasminogen (200 nmol L^−1^) was added to fibrinogen before clotting performed as described above in elastic silicon rubber tubes. After stretching, a shell filled with buffer was formed around the retracted fibrin in the rubber tube and it was replaced with 1 nmol L^−1^ tPA (Boehringer Ingelheim, Ingelheim am Rhein, Germany) using two needles pierced at the clamped ends of the tube. After incubation at 37 °C for various times, the total fluid phase was removed and its volume was measured. The concentration of plasmin in the fluid phase was determined from the enzyme activity measured on 0.1 mmol L^−1^ Spectrozyme-PL (H-D-norleucyl-hexahydrotyrosyl-lysine-p-nitroanilide; American Diagnostica, Pfungstadt, Germany) using active site-titrated plasmin with accurately known concentration as a reference (at dilutions yielding linear dependence of amidolytic activity on enzyme concentration) [12]. The amount of generated plasmin was calculated as the product of this concentration and the measured volume of the fluid surrounding fibrin and expressed in pmol per unit surface of fibrin (the area of fibrin surface after stretching was calculated as described above). In order to account for plasmin retention in fibrin, plasminogen activation was also measured in the presence of plasmin substrate Spectrozyme-PL at 0.2 mmol L^−1^ (33-fold higher concentration than its *K*_*m*_ [13]). After various incubation times, the fluid surrounding the fibrin was withdrawn and its volume and absorbance at 405 nm were measured. The amount of p-nitroaniline released from the plasmin substrate was normalized for unit surface area of the fibrin clots in the same way as the amount of plasmin.

### Release of soluble fibrin degradation products (FDP) from fibrin

Fibrin containing 200 nmol L^−1^ plasminogen was prepared as described above for monitoring plasminogen activation and fibrinolysis was initiated with 15 nmol L^−1^ tPA added to the surface. For some measurements plasminogen-free fibrin was prepared and fibrinolysis was initiated with 1 μmol L^−1^ plasmin. At 15-min intervals the fluid surrounding the fibrin was withdrawn, its volume was measured and ice-cold ethanol was added at 20% (v/v) final concentration. After centrifugation at 20 000 × *g* for 5 min, the protein content of the supernatant was determined from the values of its absorbance at 280 nm (A_280_ = 1.6 corresponds to 1 g L^−1^ non-clottable fibrin degradation products measured under identical conditions [14]).

### Morphometric analysis of fibrin structure and statistical procedures

The SEM images of thrombi and fibrin were analyzed to determine the diameter of the fibrin fibers and area of the fibrin network pores using self-designed scripts running under the Image Processing toolbox v. 7.0 of Matlab 7.10.0.499 (R2010a) (The Mathworks, Natick, MA, USA) [15]. For the diameter measurements a grid was drawn over the image with 10–15 equally-spaced horizontal lines and all fibers crossed by them were included in the analysis. The diameters were measured by manually placing the pointer of the Distance tool over the endpoints of transverse cross-sections of 300 fibers from each image (always perpendicularly to the longitudinal axis of the fibers). Pores of the gels were identified with a boundary tracing algorithm of the Image Processing Toolbox working on the whole area of the image as a region of interest. With this approach the area of the plane projections of the gel pores was measured and these values were used as dimensionality-reduced indicators of the pore size. The distribution of the data on fiber diameter and pore area was analyzed using the algorithm described previously [16]: theoretical distributions were fitted to the empirical data sets and compared using Kuiper’s test and Monte Carlo simulation procedures. The statistical evaluation of other experimental measurements in this report was performed with the Kolmogorov–Smirnov test (Statistical toolbox 7.3 of Matlab).

## Results

**Fig. 1:**
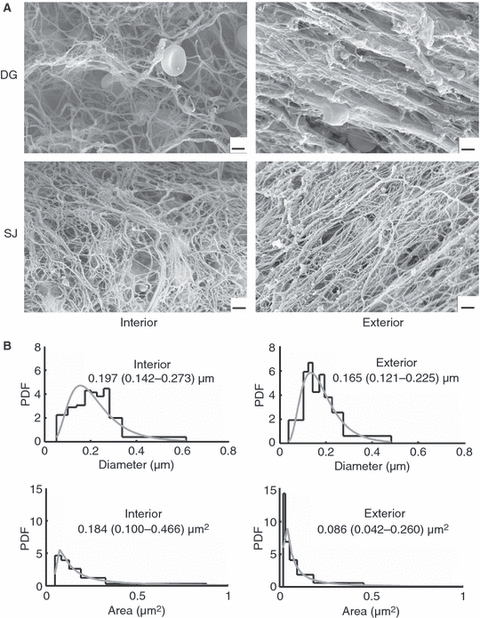
Fibrin structure on the surface and in the core of thrombi. (A) After thrombectomy thrombi were washed, fixed and dehydrated as detailed in Methods. Scanning electron microscopic (SEM) images were taken from the surface and transverse section of the same thrombus sample, scale bar = 2 μm. DG: a thrombus from popliteal artery, SJ: a thrombus from aorto-bifemoral by-pass Dacron graft. (B) Fiber diameter (upper graphs) and fibrin pore area (lower graphs) were measured from the SEM images of the DG thrombus shown in (A) using the algorithms described in Methods. The graphs present the probability density function (PDF) of the empiric distribution (black histogram) and the fitted theoretical distribution (gray curves). The numbers under the location of the observed fibrin structure show the median, as well as the bottom and the top quartile values (in brackets) of the fitted theoretical distributions. The parameters of the fitted distributions differ between the interior and exterior data sets at *P*< 0.01 level according to Kuiper’s test-based evaluation as described in Methods.

SEM images were taken from the surface and interior core regions of surgically removed thrombi in order to evaluate fibrin architecture at a microscopic scale in relation to the exposure of shear stress (Fig. 1A). In four (two in Dacron grafts, one in popliteal artery and the single pulmonary embolus) out of the 10 examined thrombi a significant difference could be observed in the arrangement of fibers along separate spatial dimensions in the interior and exterior regions of the clot. In all cases the core of the thrombi contained fibrin forming a random 3D network, whereas in four thrombi the gel pores on the surface were elongated in one direction resulting in longitudinal alignment of the fibers accompanied by their tighter packing in the transverse direction (in the remaining six cases the surface of the clot appeared similar to the core). In addition, morphometric analysis of the fibrin structure (Fig. 1B) showed that both fiber diameter and gel pore area decreased significantly (by about 16% and two-fold, respectively). These alterations of fibrin ultrastructure on the surface can be attributed to the action of the shear forces in the stenotic vessels, which in some cases are strong enough to modify mechanically the fibrin architecture.

**Fig. 2:**
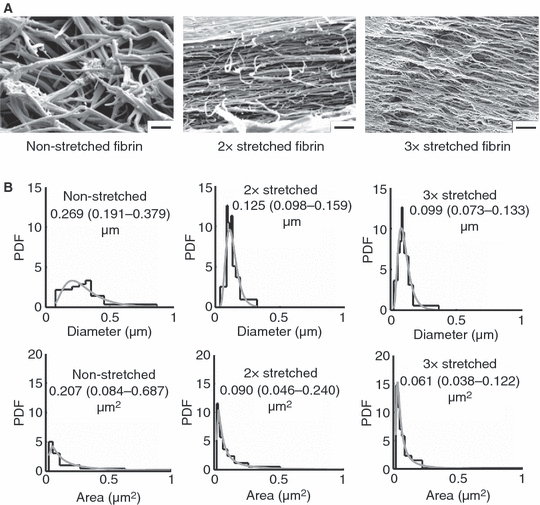
Changes in fibrin network structure caused by mechanical stretching. (A) Scanning electron microscopic (SEM) images of fibrin clots prepared from 30 μmol L^−1^ fibrinogen clotted with 30 nmol L^−1^ thrombin. Fibrin samples were fixed with glutaraldehyde before stretching or after two- and three-fold stretching as indicated, scale bar = 2 μm. (B) Fiber diameter (upper graphs) and fibrin pore area (lower graphs) were measured from the SEM images illustrated in (A) using the algorithms described in Methods. The graphs present the probability density function (PDF) of the empiric distribution (black histogram) and the fitted theoretical distribution (gray curves). The numbers under the fibrin type show the median, as well as the bottom and the top quartile values (in brackets) of the fitted theoretical distributions. The parameters of the fitted distributions differ between any two data sets at *P*< 0.001 level according to Kuiper’s test-based evaluation as described in Methods.

Because the fibrin appearance on the surface of thrombi was reminiscent of the fibrin structure reported recently for clots exposed to mechanical stretching [6], stretched clots were used (Fig. 2) as a model system to evaluate the impact of these structural alterations on the lytic susceptibility of fibrin. Stretching changed the arrangement of the fibers (Fig. 2A) to a pattern similar to the one observed on the surface of thrombi (Fig. 1A); both the median fiber diameter and the pore area of the fibrins decreased two- to three-fold and the distribution of these morphometric parameters became more homogeneous (Fig. 2B).

**Fig. 3:**
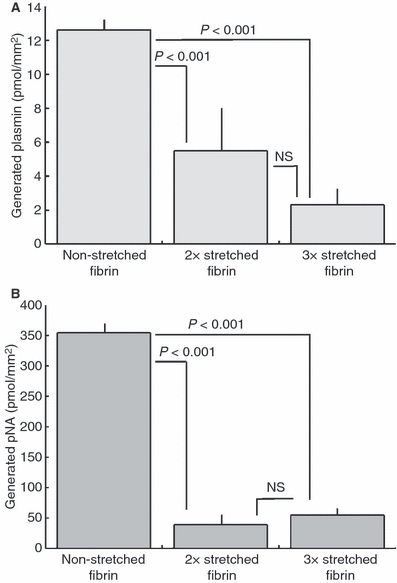
Plasminogen activation on the surface of fibrin. (A) Plasminogen (200 nmol L^−1^) was added to fibrinogen before clotting performed as in Fig. 2. After stretching, the buffer around the retracted fibrin in the rubber tube was replaced with 1 nmol L^−1^ tissue-type plasminogen activator (tPA) and after 30-min incubation at 37 °C the plasmin activity in the fluid phase was measured on 0.1 mmol L^−1^ Spectrozyme-PL. Using a series of accurately known plasmin concentrations as a reference, the amount of generated plasmin is shown (normalized for unit surface area of the fibrin clots as described in Methods). (B) Plasminogen activation was initiated under the same conditions as in (A), but the tPA solution contained 0.2 mmol L^−1^ Spectrozyme-PL. After 150-min incubation the fluid surrounding the fibrin was withdrawn and its volume and absorbance at 405 nm were measured. The amount of p-nitroaniline released from the plasmin substrate is shown (normalized for unit surface area of the fibrin clots as described in Methods). Data are presented as mean and SD (*n*= 6–9), the *P*-values refer to Kolmogorov–Smirnov test for the linked pairs of data sets (NS indicates *P*> 0.05).

**Fig. 4:**
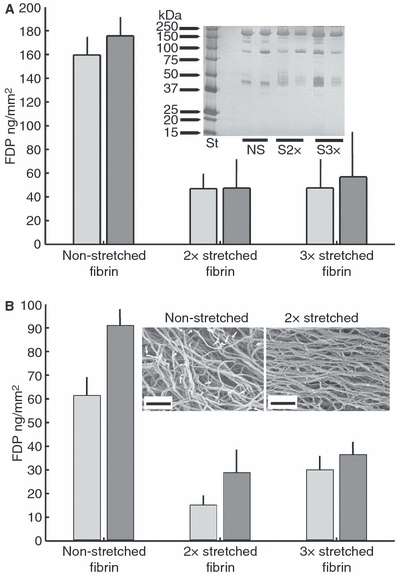
Release of soluble fibrin degradation products (FDP) from the surface of clots. (A) Fibrin containing 200 nmol L^−1^ plasminogen was prepared as in Fig. 3 and fibrinolysis was initiated with 15 nmol L^−1^ tissue-type plasminogen activator (tPA). (B) Plasminogen-free fibrin was prepared as in Fig. 2 and fibrinolysis was initiated with 1 μmol L^−1^ plasmin. At 15-min intervals the fluid surrounding the fibrin was withdrawn and its ethanol-soluble FDP content was measured as described in Methods. The amount of released FDP is shown (normalized for unit surface area of the fibrin clots) for the 1^st^ (light gray bars) and 3^rd^ (dark gray bars) 15-min period of the lysis. Data are presented as mean and SD (*n*= 4) and the differences between the non-stretched and stretched fibrins are significant at the *P <*0.01 level according to the Kolmogorov–Smirnov test. (Inset A) After adjustment for protein concentration the samples in (A) were subjected to SDS electrophoresis on 12.5% polyacrylamide gel under non-reducing conditions and silver-stained. (Inset B) After withdrawal of the fluid phase after 45-min digestion the samples in B were fixed in glutaraldehyde and SEM images were taken as described in Methods; truncated fibers are indicated by white arrows, scale bar = 2 μm.

Stretched fibrin was a poor template for plasminogen activation by tPA. The amount of plasmin generated by tPA on the surface of fibrin and released in the fluid phase decreased two- to three-fold, if stretched fibrin was used as a template instead of its non-stretched counterpart (Fig. 3A). When plasminogen activation was evaluated in the presence of a low-molecular-weight plasmin substrate, which can penetrate into the clot, the detected plasmin activity was similarly lower on stretched fibrin (Fig. 3B). Thus, the effect of the modified fibrin structure on the apparent plasmin generation is based on changes in plasminogen activation rather than in plasmin retention in the clot. In agreement with the conclusion for restricted tPA-dependent plasminogen activation on the surface of stretched fibrin detected with synthetic plasmin substrate, the non-stretched fibrin lysed completely in the time range of 65–70 min, whereas the stretched clots were observed to fracture only after 80 min into large fragments that remained visible for at least 60 min more. The release of soluble FDP from stretched fibrin clots was also slower (Fig. 4A). However, this assay measures the activity of the generated plasmin on fibrin substrates of different structure (Fig. 2) and thus the FDP release reflects changes not only in plasminogen activation, but in susceptibility of fibrin to plasmin too. In order to evaluate separately the second step (the direct fibrin solubilisation by plasmin), plasminogen-free fibrin clots were treated with plasmin and the course of their dissolution was monitored (Fig. 4B). The SEM images of non-stretched plasmin digested for 45 min with plasmin show many truncated fibers in the remnant fibrin, whereas only few fibers bear signs of digestion in the stretched fibrin (Fig. 4B, Inset). These experiments confirm that FDP release from stretched fibrin was slower but the effect was weaker than in the case of tPA-induced fibrinolysis. These results indicate that the stretched fibrin structure hinders both stages of fibrinolysis, plasminogen activation and fibrin lysis. In spite of the differences in the time-course of fibrinolysis, the molecular-size pattern of FDP released from different fibrins was essentially identical (Fig. 4A, Inset).

**Fig. 5:**
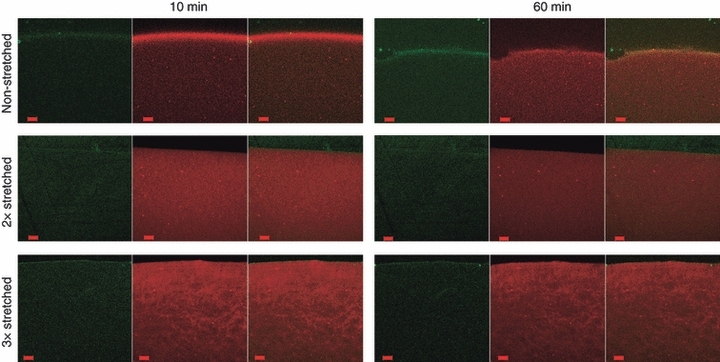
Lysis of fibrin monitored with confocal laser microscopy. Fibrin clots were prepared from 30 μmol L^−1^ fibrinogen containing 50 nmol L^−1^ Alexa^546^-labeled fibrinogen and 200 nmol L^−1^ plasminogen, clotted with 30 nmol L^−1^ thrombin and stretched as indicated. Thereafter 60 nmol L^−1^ tissue-type plasminogen activator (tPA)- green fluorescent protein (GFP) was added to fibrin and the fluid/fibrin interface was monitored with a confocal laser scanning microscope using dual fluorescent tracing: green channel for tPA and red channel for fibrin (the third panel in each image presents the overlay of the green and red channels), scale bar = 50 μm. The time after addition of tPA-GFP is indicated.

The mechanism of fibrinolytic resistance induced by stretched fibrin was approached with a recombinant fluorescent derivative of tPA (tPA-GFP) (Fig. 5). When tPA-GFP was applied to the surface of non-stretched fibrin, a distinct zone of tPA accumulation was formed at the fluid/fibrin interface within several minutes, which moved a distance of about 75 μm in 50 min as plasmin was formed and it dissolved the fibrin. The interfacial tPA-enriched zone was definitely less sharp and of smaller depth on the surface of stretched fibrin and strikingly it did not move at all in the first hour of observation. Thus, the modified ultrastucture of fibrin in clots exposed to mechanical stress impedes tPA binding/penetration into fibrin and consequently delays the lytic process in this experimental setup.

## Discussion

Stenosis of a blood vessel profoundly changes the rheological conditions around the obstruction. In addition to a several-fold increase of shear rate [7], the mechanical forces (radial, axial and circumferential) acting on the vessel wall show a heterogeneous pattern of relative strength at different locations of the stenotic region (stenosis throat, pre- and post-stenotic shoulder), but in all cases the axial force is two- to three-fold stronger than the radial force [17]. Thus, if thrombi are formed at stenotic sites of blood vessels, the fibrin fibers on their surface will be exposed to enhanced shear stress with well-defined directionality, which leads to the prediction of longitudinal alignment of these fibers. Our *ex vivo* exploration of the ultrastructure of fibrin at different locations of surgically removed thrombi provides some evidence to support this prediction (Fig. 1A). In 40% of the examined thrombi the surface fibers were aligned along one preferred axis and closer together in the perpendicular direction, whereas the fibrin meshwork in the interior parts showed random arrangement in all three dimensions of space. Although in these thrombi the individual surface fibers became thinner compared with the core of the thrombi, they formed rough bundles because of the smaller inter-fiber pore size (Fig. 1B). Our SEM data of oriented fibers at the thrombus/blood interface extend the recent report on longitudinal alignment of fibrin bundles of about 20 μm diameter observed in coronary thrombi and aortic aneurysms at the lower resolution of polarized light microscopy [18]. The surface localization of the oriented fibers reported here resolves the issue of the organizational factor for this alignment (shear stress vs. structural elements of the blood vessel wall) in favour of the flow-related forces. The fact that such a striking difference between the exterior and interior fibrin architecture was not observed in the majority of the sampled thrombi can be attributed to the variability in the magnitude of the shear stress, to which they were exposed in the blood vessels. Elaborate mathematical modeling [17] shows that the mechanical forces differ significantly even at different points of a single stenotic site, whereas the thrombi evaluated in the present study were derived from different anatomical locations and from patients with rather heterogeneous clinical background. Thus, the rheological conditions and consequently the local shear stress presumably differed significantly at the time of thrombus formation.

These ultrastructural findings raised the question about the impact of the altered fibrin architecture on the clot susceptibility to lysis. It has been well documented that thinner individual fibers are lysed more easily than thicker ones [19–22]. However, the macroscopic lytic rate does not automatically follow the trend of the individual fibers; so that in parallel with the faster individual fiber lysis evidence is provided for slower dissolution of clots composed of thin fibers in a dense conformation compared with clots composed of thicker fibers in a more open arrangement [20]. Such discrepancies can be accounted for by differences in tPA binding [23] and permeation [24] in clots of various compactness. Thus, the lytic susceptibility of the fibrin structures in thrombi reported in the present study needs to be addressed with an adequate *in vitro* model. The recently described structural properties of mechanically stretched fibrin clots [6] appeared to resemble the orientation and lateral packing of the surface fibers observed in some thrombi (Fig 1A). The appropriateness of stretched fibrin as a model of these observed structures was verified by the analogous changes in fiber diameter and gel pore area (Figs 1B and 2B). The application of stretched fibrin as a model system for the evaluation of the modifications in lytic susceptibility caused by mechanical stress has the advantage that these structures have been quantitatively characterized in terms of supra- and sub-molecular morphology and extensibility [6]. Thus, the reported geometric parameters could be used directly in our calculations of area-normalized rates of plasmin generation and fibrin dissolution. Although stretching of pre-formed fibrin results in similar fibrin architecture to the morphology observed on the surface of thrombi, the *in vivo* mechanism might be different. Two recent reports evidence that similar fibrin fiber alignment can be observed if fibrin polymerizes under flow [25,26]. Independently of the formation mechanism, however, the identical final structure supports the adequateness of the applied model for assessment of the lytic susceptibility of fibrin exposed to shear forces either at the stage of clotting or later.

Our data evidence that the stretched conformation of the clots is resistant to both tPA- and plasmin-induced lysis (Fig. 4). In spite of the reported changes in the conformation of individual fibrin monomers [6] no essential differences in the molecular-weight pattern of FDP from stretched and non-stretched fibrins could be observed (Fig. 4A, Inset). Importantly, tPA-induced fibrinolysis was apparently more sensitive to the effects of mechanical stress than the direct digestion of fibrin by plasmin (Fig. 4A vs. B) in line with additivity of altered plasminogen activation on the surface of stretched fibrin (Fig. 3) and modified plasmin susceptibility of the clot. Monitoring of the remnant fibrin clot on a microscopic scale showed that the movement of the tPA-enriched lytic front was completely blocked in stretched fibrin in the first hour of lysis (Fig. 5). In the experiments with a fluorescent chimera variant of tPA both the fluorescence intensity and the depth of the interfacial layer of tPA accumulation were smaller in the stretched fibrins suggesting weaker binding and impeded permeation as the mechanism of impaired fibrinolysis with this activator. Thus, taking a coherent view of ultrastructural and activity assays we conclude that mechanical stress, which results in higher density of re-oriented fibrin fibers confers lytic resistance related to both impaired plasminogen activation on the surface of the denser fibrin network and reduced rates of fibrin lysis by plasmin. The previously described unfolding of individual monomers in stretched fibrin [6] might possibly contribute to the hindered lysis through exposure of hydrophobic regions and expulsion of water with consequent blocking of tPA-binding and plasmin-cleavage sites in fibrin.

Based on the similarities in ultrastructure, we have correlated the lytic properties of stretched fibrin to the physical changes induced by shear stress on the surface of thrombi, but our findings may have some broader implications arising from alternative sources of mechanical stress. It is well known that platelets cause clot retraction and in the vicinity of platelets the fibrin network is oriented, denser and more resistant to lysis [3]. When the platelet content of 10 mL of blood is compacted in 400 μL of arterial thrombi [27], clots experience a large amount of mechanical strain. It has long been known that retracted clots are very resistant to fibrinolysis [23,28,29], and this resistance has been correlated with impaired tPA binding and expulsion of plasminogen. Our results gained in stretched fibrin extend the previously known factors contributing to the lytic resistance of peri-platelet zones of thrombi exposed to contractile forces of cellular origin. Together, our present findings point to the need to appreciate the role of biomechanical and rheological factors in the variable therapeutic response of patients treated with thrombolysis.

## Supporting Information

**Figure S1.** Mechanical stretching of fibrin.
