# Supplementary material for: Hindered dissolution of fibrin formed under mechanical stress
Source: J Thromb Haemost. 2011 May;9(5):979–86. doi: 10.1111/j.1538-7836.2011.04203.x (PMC3093023; doi:10.1111/j.1538-7836.2011.04203.x)

Supplementary figure

Mechanical stretching of fibrin

Thrombin was added at 30 nmol L^{-1} to fibrinogen at 30 μmol L^{-1} and immediately transferred into elastic silicon rubber tubes allowing clotting at 37 °C for 30 min. The figure illustrates the 3-fold stretching of 1.5 cm long pieces of the rubber tubes (black line) with fibrin inside. Stretching reduces the volume of fibrin, whereas the volume of the silicon mould is not changed. The fluid chamber formed around the fibrin was then used to add plasminogen activator and measure plasmin activity as described in the main text.

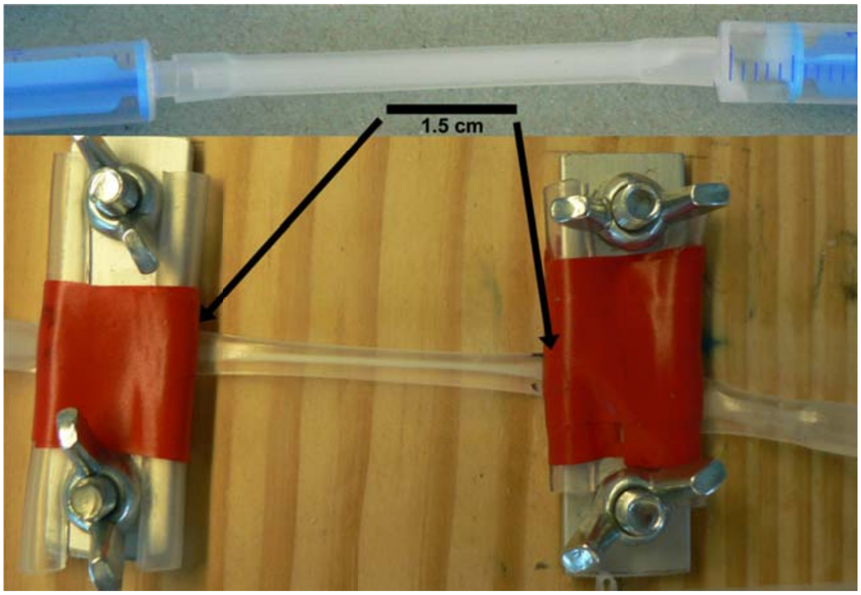

Supplement: Supplementary file 1 [file jth0009-0979-SD1.pdf]
